# Association of Race/Ethnicity, Socioeconomic Status, Acculturation, and Environmental Factors with Risk of Overweight Among Adolescents in California, 2003

**Published:** 2008-06-15

**Authors:** Min Kyung Ahn, Hee-Soon Juon, Joel Gittelsohn

**Affiliations:** Department of International Health, Bloomberg School of Public Health, Johns Hopkins University; Department of Health, Behavior, and Society, Bloomberg School of Public Health, Johns Hopkins University, Baltimore, Maryland; Department of International Health, Bloomberg School of Public Health, Johns Hopkins University, Baltimore, Maryland

## Abstract

**Introduction:**

Little has been published about racial/ethnic differences in the prevalence of overweight among adolescents that accounts in detail for socioeconomic status, acculturation, and behavioral and environmental factors. Increased understanding of factors associated with overweight can provide a rational basis for developing interventions to address the obesity epidemic in the United States.

**Methods:**

Using a cross-sectional analysis of data from adolescents who participated in the California Health Interview Survey 2003, we estimated the prevalence of overweight and at risk of overweight, combined as a single measure (AROW, body mass index ≥85th percentile). We used logistic regression models to examine associations between AROW and risk factors.

**Results:**

Twenty-nine percent of California adolescents were AROW. The prevalence of AROW differed significantly by sex and race. Boys were more likely than girls to be AROW (33% vs 25%). American Indians/Pacific Islanders/others (39%) were at highest risk, followed by Hispanics (37%), blacks (35%), whites (23%), and Asians (15%). For boys, older age, Hispanic or American Indian/Pacific Islander/other race/ethnicity, lower education of parents, and longer residence in the United States were significantly associated with AROW. For girls, Hispanic or black race/ethnicity, lower education of parents, and poor dietary habits were significantly associated with AROW.

**Conclusion:**

The high prevalence of AROW among California adolescents in most racial/ethnic groups indicates the need for culturally specific and appropriate interventions to prevent and treat overweight.

## Introduction

Overweight is epidemic among children and adolescents in the United States (1,2). Preventing overweight in children and adolescents is a public health priority because of the well-documented adverse health effects of overweight (3,4) — both short-term consequences, such as cardiovascular risk factors, asthma, and obstructive sleep apnea, and long-term consequences, such as diabetes, cardiovascular disease, hypertension, and social and economic disadvantages in adulthood (3). Overweight during childhood and adolescence also has been associated with psychosocial problems, such as poor self-image, eating disorders, and poor quality of life (3,4).

Approximately one-third of U.S. children and adolescents aged 2–19 years are either overweight (body mass index [BMI] ≥95th percentile of the reference population) or at risk of overweight (BMI ≥85th to <95th percentiles) according to the National Health and Nutrition Examination Survey (1). People of low socioeconomic status (SES) and in racial/ethnic minority groups are disproportionately affected. Studies of children and adolescents have reported significant differences among racial/ethnic groups in the prevalence of overweight (1,2,5,6). Although researchers have proposed differences in SES (such as parents' education and family income) as the main causes of racial/ethnic disparities in prevalence of overweight, in-depth data that explain the underlying causes are limited (6-8). Other factors such as demographics (e.g., age, sex) (1), acculturation (e.g., length of residence in the United States) (9,10), body image (11-14), health behaviors (e.g., dietary habits, physical activity and inactivity) (15,16), and environmental factors (e.g., physical activity facilities, prevalence of food stores, neighborhood safety) (17-21) may contribute to racial/ethnic disparities in the prevalence of overweight.

Overweight is a result of increased energy intake and physical inactivity, both of which are influenced by social, economic, and physical environments (6,18,22). Individual-level behaviors are affected by both personal characteristics and interactions with the larger social, cultural, and environmental contexts in which children and adolescents live (23). Research on predictors of childhood and adolescent overweight has focused primarily on the characteristics of children, adolescents, and parents and has not considered the context in which risk factors emerge. Researchers increasingly examine how the built environment (e.g., home, school, community) increases adolescents' risk of overweight by discouraging healthy nutrition and physical activity (14,17-20). We adopted a broader approach and examined risk factors — age, race/ethnicity, SES, acculturation, and behavioral and environmental factors — to learn about and intervene against adolescent overweight.

To our knowledge, few national studies of differences in the prevalence of overweight among large racially/ethnically diverse samples have considered in detail SES, acculturation, and behavioral and environmental factors. California's racially/ethnically diverse population provides an opportunity to examine racial/ethnic minorities that may not be commonly found in other areas. The California Health Interview Survey (CHIS) is unique in providing a large representative sample of adolescents from various racial/ethnic groups and thus a basis for clarifying patterns of adolescent overweight.

Our study addressed two goals by using a cross-sectional analysis of data from CHIS. First, we examined rates of overweight and at risk of overweight (combined as a single measure, AROW, for the purposes of this analysis) among California adolescents from various racial/ethnic groups. Second, we examined ethnicity, SES, acculturation, and behavioral and environmental factors associated with AROW among adolescents. A greater understanding of factors associated with overweight will provide a rational basis for the design and implementation of interventions to prevent obesity among U.S. adolescents.

## Methods

### Survey design and study sample

The survey focused on public health topics such as medical history, lifestyle practices, health insurance coverage, and access to health care services. CHIS is conducted every 2 years, and CHIS 2003 was the second survey. CHIS 2003 was administered through a 2-stage, geographically stratified random-digit–dialing telephone survey of California households designed to generate reliable estimates for the entire state (24). From August 2003 through February 2004, a total of 54,580 people were interviewed from 42,000 randomly selected households in every county; 4010 interviewees were adolescents aged 12–17 years (24).

The survey was administered in English, Spanish, Cantonese, Mandarin, Vietnamese, Korean, and Khmer (24). Linguistic adaptation of the survey was intended to increase the population base by including groups with limited or no English skills, which constitute a large portion of California's population. We analyzed the CHIS 2003 adolescent data public release file (25).

### Outcome and independent variables

During the telephone interview, adolescents reported their height and weight. We calculated BMI as a measure of adolescent adiposity (26). We defined "overweight" as BMI ≥95th percentile and "at risk of overweight" as BMI ≥85th but <95th percentile for age and sex according to the 2000 growth charts of the Centers for Disease Control and Prevention (27). Because the pattern and direction of results were similar in both groups, we combined the overweight and at risk of overweight categories in our statistical analysis into a single category (AROW, defined as BMI ≥85th percentile) so we could understand the full range of overweight during adolescence.

We chose independent variables on the basis of previous research and their statistical relationship with dependent variables. Our main independent variables were race/ethnicity, measures of SES, acculturation, health behavior, and physical environment. We categorized race/ethnicity on the basis of self-report as white (reference), black, Hispanic, Asian, or American Indian/Pacific Islander/other. Our combined measure of SES included both parents' education and income. We defined parents' education as the highest level of education achieved by either parent, categorized as less than high school graduate, high school graduate, some college, and college degree and/or graduate or professional degrees (reference).

We classified family income according to the U.S. Census Bureau's poverty threshold, which varies with the number and ages of family members and is revised annually to account for inflation (28). We grouped income as a dichotomous variable as follows: <100% of the federal poverty threshold or ≥100% of the federal poverty threshold (reference). As a proxy measure of acculturation, we examined an adolescent's length of residence in the United States and language spoken at home. However, because the two variables were highly associated (χ^2^ = 147.9, *P* < .001) (data not shown), we included only length of residence in the United States in the final regression model. To examine regular physical activity (coded as yes or no), we assessed survey questions to participants whether 1) in the past 7 days they had done any physical activity for at least 20 minutes on 3 or more days that made them "sweat or breathe hard" or 2) in the past 7 days, they had done any physical activity for at least half an hour on 5 days or more that did not make them "sweat or breathe hard."

To examine dietary habits, we assessed the survey questions on consumption of fruits and vegetables, fast food, and sodas or sweetened drinks during a 1-day period. The questionnaire asked, "Did you eat 5 servings of fruit and vegetables yesterday?" (respondents answered yes or no); "How many times did you eat fast foods yesterday?" (respondents gave a numerical value); and "How many times did you drink soda or sweetened drinks yesterday?" (respondents gave a numerical value). We created a new variable that accounted for all 3 of the above questions by assigning 0 to respondents who ate 5 servings of fruits and vegetables and 1 to respondents who did not. To this value we added the number of times respondents reported consuming fast food or soda and sweetened drinks. Thus, respondents with bad dietary habits would have a higher numerical score than would respondents with good dietary habits. Scores of ≤1, 2–4, and 5–24 were categorized as good, moderate, and bad dietary habits, respectively. We also assessed the effect of participants' school and community environment by examining the presence of soft drink vending machines at school (coded as yes or no) and presence of a park or playground within walking distance of home (coded as yes or no).

### Statistical analysis

Using weighted logistic regression models, we examined factors associated with AROW (BMI ≥85th percentile) as a dichotomous dependent variable. All regression analyses were conducted separately for boys and girls. We managed and analyzed data with SPSS version 14 (SPSS, Inc, Chicago, Illinois) and SUDAAN version 9.0 (Research Triangle Institute, Research Triangle Park, North Carolina). All models were weighted to allow generalization of the results to the civilian, noninstitutionalized population of California, and standard errors were corrected for survey design effects of multiple stages of cluster sampling. Standard errors were calculated with SUDAAN version 9.0 by using the jackknife method. *P* < .05 was considered statistically significant. Independent variables were chosen on the basis of previous research findings and their statistical relationships with the dependent variables. We tested colinearity by examining the variance inflation factor. No colinearity was detected because none of the variance inflation factors were greater than 10. Analyses included tests of 2-way interaction terms in regression equations.

## Results

The 4010 adolescent respondents (Table 1) represent an estimated 3,259,771 adolescents in California. Approximately half of the respondents were boys. Mean age of respondents was 14.5 years. Whites were the largest racial/ethnic group, followed by Hispanics, Asians, blacks, and American Indians/Pacific Islanders/others. Twenty percent of respondents lived below the federal poverty threshold, and the parents of 23% had less than a high school diploma. U.S.-born citizens constituted 85% of survey respondents. Approximately 55% of respondents spoke only English at home. Approximately 71% regularly participated in vigorous or moderate physical activity, and 77% attended schools that had soft drink vending machines. Most had a park or playground within walking distance of home.

Approximately 29% of surveyed adolescents were AROW. American Indians/Pacific Islanders/others were at highest risk, followed by Hispanics, blacks, whites, and Asians (Table 2). When we controlled for race/ethnicity, the prevalence of AROW differed by sex (χ^2^ = 16.2, *df* = 1, *P* < .001): boys were more likely than girls to be AROW. For whites and Hispanics, but not for other racial/ethnic groups, risk of overweight differed by sex. White boys were more likely to be AROW than were white girls (χ^2^ = 13.9, *df* = 1, *P* < .001). Hispanic boys were at higher risk than Hispanic girls (χ^2^ = 4.4, *df* = 1, *P* = .039).

In the multivariate model, age, race/ethnicity, parents' education, and acculturation were associated with AROW among boys (Table 3). Older adolescents in high school were less likely than younger adolescents in middle school to be AROW. Both Hispanic and American Indian/Pacific Islander/other adolescents were more likely than white adolescents to be AROW. Boys whose parents had less than a high school diploma or some college education were also more likely to be AROW than were adolescents whose parents had college degrees. Boys who had lived in the United States for 10 or more years or who were born in the United States were more likely to be AROW than were boys who had resided in the United States for fewer than 10 years. Family income, dietary score, physical activity, presence of a park or playground within walking distance of home, and presence of a soft drink vending machine at school were not significantly associated in the multivariate models with AROW among boys.

In the multivariate model, both Hispanic girls and black girls were significantly more likely than white girls to be AROW (Table 3). Girls whose parents had less than a high school diploma, a high school diploma, or some college were more likely to be AROW. Girls who maintained either a moderate or a bad diet were more likely than were girls who maintained a good diet to be AROW. Age, family income, acculturation, physical activity, presence of a park or playground within walking distance of home, and presence of soft drink vending machines at school were not significantly associated with AROW among girls.

## Discussion

Our study confirms earlier findings of substantial rates of overweight among U.S. adolescents (1,2). The lower prevalence of AROW in our study sample than in other nationally representative samples that used the same study definitions (1) could be due to differences in the prevalence of AROW in our population or to potential biases associated with self-report.

Our study is distinctive in its inclusion of a large number of Asian and Pacific Islander/American Indian/other adolescents. We could estimate prevalence of AROW in these populations with considerable confidence and present corresponding estimates for a segment of the U.S. population that many studies and national surveys have omitted.

Reasons are complex for the significant differences in the prevalence of AROW among racial/ethnic groups. Previous research suggests SES, acculturation, and sociocultural beliefs and practices may be related to the variation. Differing attitudes toward ideal body size also might explain racial/ethnic differences. In studies of adolescent girls, blacks preferred a significantly heavier ideal body size than did whites and were more likely than whites to be satisfied with their body size, to describe themselves as thinner than other girls, and to say they were not overweight (12,13). Although Hispanic adolescents may report levels of body dissatisfaction similar to those of white adolescents, they are more likely to rate themselves as attractive and to report more positive attitudes toward obesity (11,14). In addition, white high school girls report being more likely than blacks and Hispanics to exercise to lose or maintain their weight (2).

The higher risk of overweight in boys than in girls probably results from biological and behavioral differences. The Youth Risk Behavior Survey indicated significant differences by sex in adolescents' dietary behaviors and physical activity patterns (2); boys were more likely than girls to watch television and less likely to exercise to lose weight or to avoid gaining weight (2).

We found an association between acculturation and prevalence of AROW among boys. Consistent with findings from earlier studies (9,10), immigrant boys who had resided longer in the United States or boys who were born in the United States were at greater risk of overweight than were more recent U.S. immigrants. Lifestyle differences between foreign-born and U.S.-born adolescents most likely account for the differences. Over time, immigrant children adopt American behavioral norms for health status and risk, including sedentary lifestyles, large portion sizes, and consumption of high-fat, energy-dense foods. Acculturation during adolescence may be especially intense given the influence of peers on adolescents.

For both sexes, adolescents whose parents had a lower level of education were more likely to be AROW. In contrast, family income was not significantly related to risk of overweight. U.S. children and adolescents in high-SES groups generally are considered less likely to become overweight than are their low-SES counterparts (7,8). However, recent studies have challenged this view, indicating a weakening association between SES and obesity, with patterns differing by race/ethnicity (6,7,29). Parents' education may play a role in an adolescent's risk of overweight by numerous mechanisms. For example, less educated parents may know less about the role of nutrition, and parents' knowledge of nutrition may influence adolescents' eating patterns (30). Mothers' knowledge about nutrition and concern for disease prevention have been positively associated with children's fruit and vegetable intake (30) and negatively associated with children's total energy and fat intake (31). Lack of knowledge about appropriate serving sizes may cause parents to overfeed children and adolescents; serving larger portions is associated with greater food intake (32).

We did not find statistically significant associations between the environmental factors we examined and prevalence of AROW. However, because measures used in our study evidently have limitations related to our secondary analysis of data, we cannot conclude that environmental factors do not influence weight. Despite the lack of a significant relationship between soft drink vending machines at school and AROW among the adolescents we surveyed, we found that soft drink vending machines were extremely prevalent — more than three-fourths of adolescents reported their schools had them — which shows that students have easy access to sugar beverages at school. Soft drink consumption increased by 100% among U.S. adolescents during the past 2 decades (33), and soft drink consumption is associated with excess energy intake and weight gain among adolescents (16,33,34). Children and adolescents consume 35%–40% of total energy at school (35,36); therefore, nutrition intervention programs at school should account for food and beverages available in the school environment, including vending machines and school lunch programs. Despite the potential influence of vending machines at school, few qualitative and quantitative data are available on the influence of vending machines on adolescents' weight. Future research should investigate links between the school food environment, adolescents' food choices, and quality of their diet.

We found no significant associations between the presence of a park or playground within walking distance of home and risk of overweight. Studies increasingly are examining how the built environment raises children's and adolescents' risk of overweight by encouraging consumption of energy-dense foods and discouraging physical activity (17-20). However, the exact mechanisms by which environmental factors influence weight gain or dietary choices are largely speculative. The availability of recreational facilities is associated with substantially increased bouts of physical activity and decreased overweight (17). Other research is examining the availability and accessibility of both healthy and unhealthy food options within neighborhoods (18,20).

The findings of our study are subject to some limitations. First, because we based the study on cross-sectional data, we cannot infer any causal relationship. Further research should confirm our findings and examine causal mechanisms so appropriate interventions can be planned. Second, height and weight data were based on self-report and not validated by physical examination; because adolescents tend to overreport their height and underreport their weight (37,38), the data could have resulted in misclassification of AROW participants. Third, our study analyzed only risk factors measured in CHIS, a subset of a much larger pool of risk factors that cause adolescent overweight (e.g., sedentary activities such as television watching [15,39]; parental overweight [40]; perceived dangerousness of neighborhoods [21]; limited access to supermarkets that sell nutritious, low-calorie food [20]). Future research should address these factors as well. Fourth, our study explored only the availability and accessibility of a park or playground without considering the quality of recreational facilities and barriers to access, such as a crime rate that might encourage parents to keep children at home. Finally, our secondary analysis of data presented challenges in evaluating associations between certain risk factors and risk of overweight. The validity of an adolescent's dietary habit score as a measure of his or her usual dietary intake pattern was questionable because we assessed eating pattern by asking only 3 dietary intake questions about foods consumed in a 1-day period; diet could have differed significantly depending on the day (weekday or weekend) the participant was interviewed.

Ethnic disparities were substantial in the prevalence of AROW among California adolescents. The high prevalence in most racial/ethnic groups in our analysis indicates the need for culturally specific interventions to prevent and treat overweight. Future research should examine potential explanations for these disparities so appropriate interventions can be designed and implemented to address the obesity epidemic among U.S. children and adolescents.

## Figures and Tables

**Table 1:** Characteristics of Adolescent Respondents Aged 12–17 Years, California Health Interview Survey, 2003 (N = 4010)

**Characteristic**	**% (SE)^a^**
**Age, y (mean 14.46 ± 0.02)**	
12–13	34.0 (0.73)
14–17	66.0 (0.73)
**Sex**	
Male	51.2 (0.00)
Female	48.8 (0.00)
**Race/ethnicity**	
White	41.4 (0.53)
Hispanic	34.0 (0.60)
Black	9.0 (0.17)
Asian	10.2 (0.15)
American Indian/Pacific Islander/other	5.4 (0.43)
**Parents' education**	
Less than high school	23.2 (0.75)
High school	21.7 (0.94)
Some college	26.9 (1.03)
College or more	28.2 (0.83)
**Income as percentage of federal poverty threshold**	
<100%	20.1 (0.86)
100%–199%	22.2 (0.99)
200%–299%	14.6 (0.80)
≥300%	43.1 (1.00)
**Citizenship**	
U.S.-born	85.0 (0.76)
Naturalized U.S. citizen	4.8 (0.49)
Non-U.S. citizen	10.2 (0.67)
**Length of residence in United States, y**	
<5	5.0 (0.52)
5–9	3.7 (0.49)
10–14	5.0 (0.53)
≥15 or U.S.-born	86.3 (0.76)
**Language spoken at home**	
English	54.4 (0.85)
English and 1 other language	39.9 (0.86)
Other than English	5.7 (0.54)
**Regular vigorous or moderate physical activity**	
Yes	70.7 (0.98)
No	29.4 (0.98)
**Soft drink vending machines at school**	
Yes	77.0 (0.85)
No	23.0 (0.85)
**Park or playground within walking distance of home**	
Yes	81.6 (0.90)
No	18.5 (0.90)

a All SEs were adjusted for design effect with SUDAAN (Research Triangle Institute, Research Triangle Park, North Carolina).

**Table 2:** Normal Weight and AROW^a^ Among Adolescents Aged 12–17 Years by Sex and Racial/Ethnic Group, California Health Interview Survey, 2003 (N = 4010)

Racial/Ethnic Group	Boys**, % (SE)^b^**	Girls**, % (SE)^b^**	**Total, % (SE)^b^**	**Sex Difference*,*** * P* Value
**Total sample**				
Normal weight	67.6 (1.33)	75.1 (1.33)	71.2 (0.94)	
AROW	32.5 (1.10)	24.9 (1.15)	28.8 (0.82)	<.001
**White**				
Normal weight	71.3 (1.72)	81.6 (1.72)	76.9 (1.18)	
AROW	27.7 (1.55)	18.4 (1.37)	23.1 (1.01)	<.001
**Black**				
Normal weight	66.5 (4.80)	64.4 (5.62)	65.5 (3.35)	
AROW	33.5 (4.35)	35.6 (5.46)	34.6 (3.34)	.794
**Hispanic**				
Normal weight	59.1 (2.92)	68.1 (2.75)	63.4 (1.88)	
AROW	40.9 (2.14)	31.9 (2.39)	36.6 (1.58)	.039
**Asian American**				
Normal weight	83.0 (3.57)	86.6 (3.88)	84.7 (2.50)	
AROW	17.0 (3.22)	13.4 (3.17)	15.3 (2.09)	.512
**American Indian/Pacific Islander/other**				
Normal weight	56.5 (6.76)	65.5 (6.95)	61.0 (4.57)	
AROW	43.5 (6.04)	34.5 (4.44)	39.0 (3.87)	.379

a Overweight and at risk of overweight were combined into a single measure (AROW), defined as body mass index ≥85th percentile for age and sex according to the growth charts of the Centers for Disease Control and Prevention (27).

b All SEs were adjusted for design effect with SUDAAN (Research Triangle Institute, Research Triangle Park, NC).

**Table 3:** Risk of AROW^a^ Among Adolescents in California by Sex and Selected Characteristics, California Health Interview Survey 2003 (N = 4010)

Variable	Risk of AROW			

	Boys		Girls	

	Crude OR (95% CI)^b^	Adjusted OR (95% CI)^b^	Crude OR (95% CI)^b^	Adjusted OR (95% CI)^b^
**Age, y (referent, 12-13 y)**				
14–17	0.75 (0.59-0.96)^c^	0.72 (0.54-0.96)^c^	0.74 (0.55-1.00)	0.86 (0.61-1.22)
**Race/ethnicity (referent, white)**				
Hispanic	1.81 (1.38-2.38)^c^	1.52 (1.08-2.13)^c^	2.08 (1.43-3.00)^c^	1.54 (1.01-2.36)^c^
Black	1.32 (0.83- 2.11)	1.21 (0.75-1.97)	2.45 (1.46-4.12)^c^	2.11 (1.20-3.73)^c^
Asian	0.54 (0.31- 0.93)^c^	0.62 (0.35-1.09)	0.68 (0.31-1.50)	0.69 (0.32-1.50)
American Indian/Pacific Islander/other	2.02 (1.13-3.59)^c^	1.89 (1.06-3.39)^c^	2.34 (1.20-4.55)^c^	1.82 (0.93-3.54)
**Parents' education (referent, college or more)**				
Less than high school graduate	2.67 (1.86-3.84)^c^	2.07 (1.28-3.35)^c^	2.95 (1.77-4.94)^c^	2.00 (1.05-3.80)^c^
High school graduate	1.39 (0.96-2.03)	1.16 (0.79-1.71)	2.95 (1.81-4.81)^c^	2.33 (1.39-3.90)^c^
Some college	1.91 (1.33-2.75)^c^	1.72 (1.18-2.51)^c^	2.06 (1.25-3.40)^c^	1.71 (1.04-2.81)^c^
**Income as percentage of federal poverty threshold (referent, ≥100%)**				
<100%	1.44 (1.03-2.03)^c^	1.05 (0.72-1.58)	1.79 (1.25-2.56)^c^	1.28 (0.79-2.08)
**Length of stay in United States, y (referent, <10 y)**				
≥10 or U.S.-born	1.62 (0.98-2.66)	2.06 (1.22-3.47)^c^	0.90 (0.50-1.62)	1.00 (0.51-1.97)
**Dietary habit score (referent, good)**				
Moderate	1.15 (0.68-1.94)	1.08 (0.64-1.18)	2.88 (1.58-5.26)^c^	2.50 (1.33-4.71)^c^
Bad	1.02 (0.67-1.55)	0.85 (0.56-1.30)	2.48 (1.38-4.44)^c^	1.87 (1.03-3.41)^c^
**Regular moderate/vigorous physical activity (referent, yes)**				
No	1.34 (0.96-1.88)	1.33 (0.96-1.85)	1.03 (0.71-1.49)	0.94 (0.63-1.40)
**Soft drink vending machine at school (referent, no)**				
Yes	0.98 (0.70-1.36)	1.05 (0.74-1.50)	0.67 (0.50-0.88)^c^	0.71 (0.50-1.00)
**Park or playground within walking distance of home (referent, yes)**				
Yes	Referent	Referent	Referent	Referent
No	1.19 (0.85-1.68)	1.11 (0.78-1.60)	0.98 (0.66-1.47)	0.93 (0.62-1.39)

OR indicates odds ratio; CI, confidence interval.

a Overweight and at risk of overweight were combined into a single measure (AROW), defined as body mass index ≥85th percentile for age and sex according to the growth charts of the Centers for Disease Control and Prevention (27).

b 95% CIs were computed on the basis of sample weights provided by the California Health Interview Survey, 2003.

c Significant at *P* < .05.

## References

[1] Ogden CL, Carroll MD, Curtin LR, McDowell MA, Tabak CJ, Flegal KM (2006). Prevalence of overweight and obesity in the United States, 1999–2004. JAMA.

[2] Grunbaum JA, Kann L, Kinchen S, Ross J, Hawkins J, Lowry R (2004). Youth risk behavior surveillance — United States, 2003. MMWR Surveill Summ.

[3] Dietz WH (1998). Health consequences of obesity in youth: childhood predictors of adult disease. Pediatrics.

[4] Strauss RS (2000). Childhood obesity and self-esteem. Pediatrics.

[5] Baruffi G, Hardy CJ, Waslien CI, Uyehara SJ, Krupitsky D (2004). Ethnic differences in the prevalence of overweight among young children in Hawaii. J Am Diet Assoc.

[6] Gordon-Larsen P, Adair LS, Popkin BM (2003). The relationship of ethnicity, socioeconomic factors, and overweight in US adolescents. Obes Res.

[7] Wang Y, Zhang Q (2006). Are American children and adolescents of low socioeconomic status at increased risk of obesity? Changes in the association between overweight and family income between 1971 and 2002. Am J Clin Nutr.

[8] Stunkard AJ, Sørensen TI (1993). Obesity and socioeconomic status — a complex relation. N Engl J Med.

[9] Goel MS, McCarthy EP, Phillips RS, Wee CC (2004). Obesity among US immigrant subgroups by duration of residence. JAMA.

[10] Popkin BM, Udry JR (1998). Adolescent obesity increases significantly in second and third generation U.S. immigrants: the National Longitudinal Study of Adolescent Health. J Nutr.

[11] Cachelin FM Rebeck RM Chung GH Pelayo E 10 3 2002 158 166 Does ethnicity influence body-size preference? A comparison of body image and body size Obes Res Cachelin FM, Rebeck RM, Chung GH, Pelayo E. Does ethnicity influence body-size preference? A comparison of body image and body size. Obes Res 2002;10(3):158-66. 11886938 10.1038/oby.2002.25

[12] Neff LJ, Sargent RG, McKeown RE, Jackson KL, Valois RF (1997). Black-white differences in body size perceptions and weight management practices among adolescent females. J Adolesc Health.

[13] Parnell K, Sargent R, Thompson SH, Duhe SF, Valois RF, Kemper RC (1996). Black and white adolescent females' perceptions of ideal body size. J Sch Health.

[14] Robinson TN, Killen JD, Litt IF, Hammer LD, Wilson DM, Haydel KF (1996). Ethnicity and body dissatisfaction: are Hispanic and Asian girls at increased risk for eating disorders?. J Adolesc Health.

[15] Crespo CJ, Smit E, Troiano RP, Bartlett SJ, Macera CA, Andersen RE (2001). Television watching, energy intake, and obesity in US children: results from the third National Health and Nutrition Examination Survey, 1988-1994. Arch Pediatr Adolesc Med.

[16] Ludwig DS, Peterson KE, Gortmaker SL (2001). Relation between consumption of sugar-sweetened drinks and childhood obesity: a prospective, observational analysis. Lancet.

[17] Gordon-Larsen P, Nelson MC, Page P, Popkin BM (2006). Inequality in the built environment underlies key health disparities in physical activity and obesity. Pediatrics.

[18] French SA, Story M, Jeffery RW (2001). Environmental influences on eating and physical activity. Annu Rev Public Health.

[19] Hill JO, Peters JC (1998). Environmental contributions to the obesity epidemi. Science.

[20] Morland K, Wing S, Diez Roux, Poole C (2002). Neighborhood characteristics associated with the location of food stores and food service places. Am J Prev Med.

[21] Molnar BE, Gortmaker SL, Bull FC, Buka SL (2004). Unsafe to play? Neighborhood disorder and lack of safety predict reduced physical activity among urban children and adolescents. Am J Health Promot.

[22] Ebbeling CB, Pawlak DB, Ludwig DS (2002). Childhood obesity: public-health crisis, common sense cure. Lancet.

[23] Davison KK, Birch LL (2001). Childhood overweight: a contextual model and recommendations for future research. Obes Rev.

[24] (2005). California Health Interview Survey. CHIS 2003 methodology series: report 1 – sample design.

[25] (2005). California Health Interview Survey. CHIS 2003 adolescent public use file, release 1 [computer file].

[26] Dietz WH, Bellizzi MC (1999). Introduction: the use of body mass index to assess obesity in children. Am J Clin Nutr.

[27] 2000 CDC growth charts: United States.

[28] How the Census Bureau measures poverty (official measure).

[29] Zhang Q, Wang Y (2004). Trends in the association between obesity and socioeconomic status in U.S. adults: 1971 to 2000. Obes Res.

[30] Gibson EL, Wardle J, Watts CJ (1998). Fruit and vegetable consumption, nutritional knowledge and beliefs in mothers and children. Appetite.

[31] Golan M, Crow S (2004). Parents are key players in the prevention and treatment of weight-related problems. Nutr Rev.

[32] Rolls BJ, Roe LS, Meengs JS (2006). Larger portion sizes lead to a sustained increase in energy intake over 2 days. J Am Diet Assoc.

[33] Guthrie JF, Morton JF (2000). Guthrie JF, Morton JF. Food sources of added sweeteners in the diets of Americans. J Am Diet Assoc.

[34] Harnack L, Stang J, Story M (1999). Soft drink consumption among US children and adolescents: nutritional consequences. J Am Diet Assoc.

[35] Kubik MY, Lytle LA, Hannan PJ, Perry CL, Story M (2003). The association of the school food environment with dietary behaviors of young adolescents. Am J Public Health.

[36] Dwyer J (1995). The School Nutrition Dietary Assessment Study. Am J Clin Nutr.

[37] Brener ND, McManus T, Galuska DA, Lowry R, Wechsler H (2003). Reliability and validity of self-reported height and weight among high school students. J Adolesc Health.

[38] Goodman E, Hinden BR, Khandelwal S (2000). Accuracy of teen and parental reports of obesity and body mass index. Pediatrics.

[39] Gordon-Larsen P, Adair LS, Popkin BM (2002). Ethnic differences in physical activity and inactivity patterns and overweight status. Obes Res.

[40] Whitaker RC, Wright JA, Pepe MS, Seidel KD, Dietz WH (1997). Predicting obesity in young adulthood from childhood and parental obesity. N Engl J Med.

